# Glycosidic enzymes enhance retinal transduction following intravitreal delivery of AAV2

**Published:** 2011-06-30

**Authors:** Jasmina Cehajic-Kapetanovic, Magali M. Le Goff, Annette Allen, Robert J. Lucas, Paul N. Bishop

**Affiliations:** 1 School of Biomedicine, University of Manchester, Manchester Academic Health Science Centre, Manchester, UK; 2 Faculty of Life Sciences, University of Manchester, Manchester Academic Health Science Centre, Manchester, UK; 3 Manchester Royal Eye Hospital, Central Manchester University Hospitals NHS Foundation Trust, Manchester, UK

## Abstract

**Purpose:**

To determine whether the co-injection of extracellular matrix degrading enzymes improves retinal transduction following intravitreal delivery of adeno-associated virus-2 (AAV2).

**Methods:**

AAV2 containing cDNA encoding enhanced green fluorescent protein (GFP), under the control of a chicken β-actin promoter, was delivered by intravitreal injection to adult mice in conjunction with enzymes including collagenase, hyaluronan lyase, heparinase III, or chondroitin ABC lyase. Two weeks later, retinal flatmounts were examined for GFP expression using confocal microscopy.

**Results:**

Without the addition of enzymes, transduction was limited to occasional cells in the retinal ganglion cell layer. The addition of heparinase III or chondroitin ABC lyase greatly enhanced transduction of the retinal ganglion cell layer and increased the depth of transduction into the outer retina. Hyaluronan lyase had a limited effect and collagenase was ineffective. Electroretinograms survived with higher concentrations of heparinase III and chondroitin ABC lyase than were required for optimal retinal transduction.

**Conclusions:**

AAV2-mediated retinal transduction is improved by co-injection of heparinase III or chondroitin ABC lyase. Improved transduction efficiency may allow intravitreal injection to become the preferred route for delivering gene therapy to both the inner and outer retina.

## Introduction

To date, adeno-associated virus (AAV) has been the most effective vector for retinal gene delivery because it elicits minimal immune response and can mediate long-term transgene expression in a variety of non-dividing retinal cell types. AAV is a nonhuman pathogen of the *Parvoviridae* family possessing a single-stranded DNA genome (4.7 kb) with two open reading frames, *rep* (for replication) and *cap* (encodes capsid proteins), flanked by two symmetric inverted terminal repeats. Recombinant AAV vectors are generated by replacing *rep* and *cap* with the required cDNA [1].

The prototype and the most studied AAV is serotype-2 (AAV2). This has been used in clinical trials, with very encouraging results, to treat Leber congenital amaurosis by transducing the retinal pigment epithelium with RPE65 cDNA [2-4]. In addition, a variety of other AAV serotypes and hybrid forms have been shown to be capable of retinal transduction [1,5]. In most studies to date, subretinal injection has been used to deliver AAV to the retina.

This delivery method creates a temporary separation bleb between the neurosensory retina and the retinal pigment epithelium, providing gene delivery to neighboring cells. Intravitreal delivery has potential advantages over this subretinal approach because it is less technically challenging and is less prone to complications, particularly through surgical manipulation of thin degenerating retinas, which may cause retinal hemorrhage, tear or detachment. Furthermore, the intravitreal approach can potentially deliver more widespread transduction across the retina when compared to localized subretinal blebs.

Intravitreal injection has been used to transduce retinal ganglion cells and bipolar cells, but at present this approach produces relatively low efficiency retinal transduction. One potential limit to the efficacy of intravitreal viral injection stems from the physical barriers formed by the vitreous, internal limiting membrane (ILM), retinal extracellular matrix (ECM), and cell surface proteoglycans. The vitreous, ILM, and retinal ECM all contain glycosaminoglycans (GAGs), while the vitreous and ILM contain collagens. The vitreous is a highly hydrated ECM that contains GAGs and a low concentration of collagen fibrils [6]. The predominant GAG in vitreous is hyaluronan (HA), but it also contains small amounts of chondroitin sulfate proteoglycans (CSPGs). The ILM contains a basement membrane called the internal limiting lamina (ILL); this is a sheet-like extracellular matrix containing type IV collagen, laminins, nidogen-1 and 2, and heparan sulfate proteoglycans (HSPGs) that include type XVIII collagen, perlecan, and agrin [7]. The neurosensory retina contains chondroitin and heparan sulfate proteoglycans, but a mouse retina does not contain HA [8,9]. Cell surface heparan sulfate proteoglycans are present in the retina, particularly on the neurites of neuronal cells. Chondroitin sulfate proteoglycans are present in the ILM, in the nerve fiber-rich layers of the retina, which include the inner and outer plexiform layer, and in the interphotoreceptor matrix (IPM) [8].

We hypothesized that enzymatic degradation of collagen or specific GAGs would increase retinal transduction efficiency with AAV2. We therefore tested the efficacy of co-injecting extracellular matrix degrading enzymes with AAV2 into the mouse vitreous on retinal expression of a reporter gene (enhanced green fluorescent protein; GFP) contained within the viral vector. We found that both heparinase III and chondroitin ABC lyase markedly improved the efficiency of retinal transduction.

## Methods

### Experimental animals

All animal experiments were conducted in accordance with the UK Home Office regulations for the care and use of laboratory animals, the UK Animals (Scientific Procedures) Act (1986), and the ARVO Statement for the Use of Animals in Ophthalmic and Vision Research. Adult *Opn4^-/+^* and wild-type C57BL/6J mice were used for all studies. All mice were kept under a 12 h:12 h light-dark cycle and were given free access to food and water.

### Generation of rAAV vectors

The vector, rAAV serotype 2 (rAVETM), expressing GFP under the control of a chicken β-actin promoter (AAV-2.CBA.eGFP) was obtained from Genedetect, Auckland, New Zealand.

### Enzymes and dilutions

The following enzymes, all obtained from Sigma-Aldrich (Dorset, UK), were used: high purity bacterial collagenase (from *Clostridium histolyticum*, Type VII); hyaluronan lyase from *Streptomyces* (E.C. 4.2.2.1), which has a high specificity for hyaluronan; Chondroitin ABC lyase (E.C. 4.2.2.4), which cleaves chondroitin and dermatan sulfates and has some activity against hyaluronan; and Heparinase III (E.C. 4.2.2.8) which selectively cleaves heparan sulfates. All enzyme solutions were made fresh on the day of injection by dissolving the enzymes in sterile phosphate-buffered saline (PBS).

### Intravitreal injections

Prior to intravitreal injections, mice were anaesthetized with isoflurane. One drop of 1% proxymethocaine was applied topically to each eye as a local anesthesia. A fine glass micropipette connected to a 5 μl Hamilton glass syringe was passed at the equator through the sclera and into the vitreous cavity, carefully avoiding the lens. Each eye received two injections with a combined volume of 0.5 μl. The injections were administered slowly over approximately one minute. The first injection, containing 0.25 μl of 1×10^12^ genomic particles/ml of AAV-2.CBA.eGFP diluted at a ratio 1:5 with PBS (i.e., 5×10^7^ genomic particles), was followed immediately by a second 0.25 μl injection of PBS containing either zero or varying amounts of enzyme. Post procedure buprenorphine (0.1 mg/kg) was administered subcutaneously for analgesia.

### Electroretinography

Retinal function was evaluated 2 weeks after intravitreal injection of 800 or 1,600 units of chondroitin ABC lyase or heparinase III in 6 mice. Both dark- and light-adapted electroretinograms (ERGs) were recorded. Briefly, mice were dark adapted overnight and anesthetized with an intraperitoneal injection of a mixture of ketamine (75 mg/ml, 10%) and xylazine (13.6 mg/ml, 20%). The mice were placed on a heating pad during the procedure to maintain body temperature. Pupils were dilated with 1% tropicamide. A gold wire loop electrode was placed on the surface of the cornea. A reference electrode was inserted into the left cheek, a differential electrode was placed under the skin on the forehead, and a neutral electrode was inserted subcutaneously near the tail. Electrical signals were amplified using an amplifier with 10^4^× gain and a bandwidth of 0.1 to 1 kHz (–3 dB points). Signals were digitized at a rate of 5.12 kHz. ERG signals were averaged three to six times to reduce noise.

### Retina flatmounts

Two weeks after injection, the mice were euthanized and their eyes were then enucleated and fixed with 4% paraformaldehyde (PFA) for 1 h at room temperature. The lenses were removed anteriorly under a light microscope. The aphakic eyes were immersion-fixed in 4% PFA overnight at 4 °C and then washed with PBS. The entire retinas were carefully dissected from the eyecup under a light microscope, blocked with PBS containing 0.5% Triton X-100 for 1 h, and were washed again with PBS buffer. To make flatmounts, the retinas were mounted on glass slides, making four radial cuts from the edges to the equator of the retina. A drop of Vectashield (Vector Laboratories Ltd., Peterborough, UK), containing DAPI stain, was applied before coverslips were put in place.

### Cryosections

Two weeks post intravitreal injection, the mice were euthanized and their eyes were enucleated. The cornea and lens were removed anteriorly under a light microscope and tissue was fixed with 4% paraformaldehyde (PFA) for 2–3 h at room temperature. The eyecups were then washed in PBS and immersion-fixed overnight in 30% sucrose in PBS. Eyes were then embedded and frozen in an optimal-cutting temperature medium (Raymond A Lamb Ltd., Eastbourne,UK). A cryostat was used to obtain 5–10 µm sections. These sections were mounted on a glass slide and a drop of Vectashield (Vector Laboratories Ltd., Peterborough, UK) containing DAPI stain was applied before coverslips were put in place.

### Microscopy

Flatmounts were examined using a Leica SP5 inverted confocal laser scanning microscope (Argon 405 20%) with a 63× oil-immersion objective. To quantify GFP expression, serial optical z-sectioning was performed over an area of 0.056 mm^2^ on retina flatmounts and through the full thickness of the retina. All images were taken under identical conditions of laser intensity, brightness, and contrast. For analyses of total fluorescence within a given portion of retina the captured stacks of images were collapsed over the z-axis into confocal projections using Leica Microsystems LAS AF software. These z-sectioned constructs were then processed using ImageJ Software (downloaded from National Institutes of Health website).

**Figure 1:**
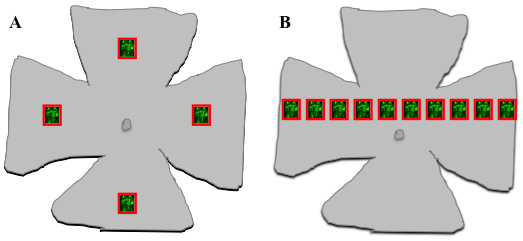
Schematic representation of the methodology used to quantify GFP expression in mouse retina flatmounts. Confocal z-sectioning (serial optical sections) was performed in areas of 238×238 μm at 1 μm intervals through the depth of the retina. All images were captured under identical conditions of laser intensity, brightness and contrast. Slice image stacks were collapsed over the z-axis into confocal projections using Leica Microsystems LAS AF software and processed with ImageJ software to measure the total (GFP mediated) fluorescence in pixels per area through the retinal depth. For initial analyses of enzymes efficacy, an area was selected at random in the mid-periphery of each quadrant (**A**), and for dose–response curves ten areas were selected along a randomly chosen straight extension across the retina (**B**). All analyses were conducted blind to the amount of enzyme used.

Briefly, GFP fluorescence intensity was quantified in pixels per area for each section in a confocal stack. The total intensity was then summed for each stack. Low level, diffuse, endogenous background fluorescence was variably present in the sections in the confocal stacks, which had a distinct appearance from the fluorescence observed in GFP transduced cells. Therefore, the threshold was adjusted for each z-construct to exclude this non-specific background fluorescence. To ensure equal starting plane depths, the retinal tissue surface (vitreal side) was first focused by using plain field light microscopy, and by then switching to the confocal laser channels. For initial assessments of enzyme activity, four randomly chosen sites in the mid-periphery of the retinal flatmounts were analyzed (Figure 1A); for enzyme dose–response curves, ten evenly spaced samples in a straight line across the retina, which were centered upon but avoided the optic nerve head, were analyzed (Figure 1B).

Retinal cryosections were analyzed under an Olympus BX51 upright microscope using a 20×/0.30 Plan Fln objective and captured using a Coolsnap ES camera (Photometrics, Tucson, AZ) through MetaVue Software (Molecular Devices Ltd. Wokingham, UK). Specific band pass filter sets for Texas Red (to identify endogenous fluorescence), DAPI (to analyze nuclei), and FITC (to analyze GFP expression), were used to prevent bleed through from one channel to the next. Images were then processed and analyzed using ImageJ.

### Statistical analysis

Twelve eyes were injected for the initial evaluation of each enzyme (three eyes per enzyme concentration) and four random retinal areas per eye were analyzed by measuring total fluorescence in collapsed z-stacks. Geometric mean fluorescence intensity with a 95% CI was calculated for each enzyme concentration. Data are presented as the percentage GFP intensity of the specified enzyme concentrations compared with the control group (0 units - i.e., AAV-2.CBA.eGFP with no added enzyme). Differences between groups were evaluated using one-way ANOVA followed by Dunnett’s post-test in GraphPad Prism. Significance was set at p<0.05.

For enzyme dose–response curves (chondroitin ABC lyase and heparinase III), three eyes were injected per enzyme concentration and 10 retinal areas per eye were examined as described above (Figure 1B). The geometric mean (geomean) of these repeated measures was then calculated for each eye to estimate the fluorescence for subsequent analysis and presentation.

## Results

### Glycosidic enzymes enhance retinal transduction

**Figure 2:**
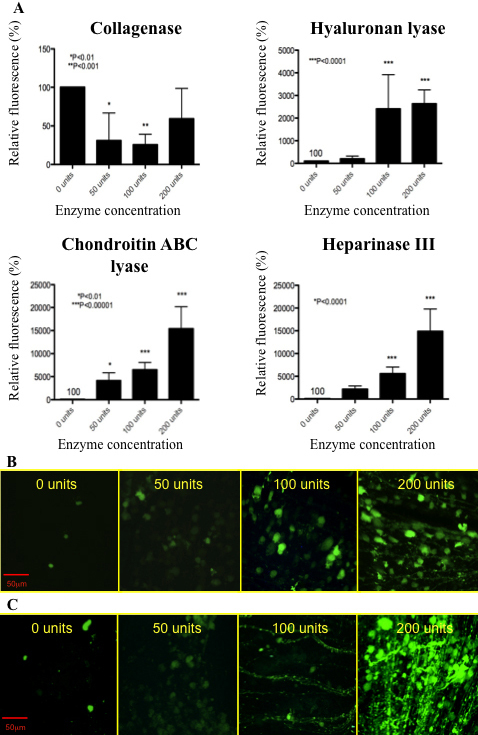
Retinal fluorescence two weeks after the intravitreal delivery of AAV2.CBA.eGFP and other different enzymes. **A**: Quantitative analysis of fluorescence following treatment with the AAV2 and collagenase, hyaluronan lyase, chondroitin ABC lyase, or heparinase III in increasing concentrations. Data are geomeans with a ±95% CI (n=9 eyes), and show the percentage fluorescence intensity relative to that induced by AAV2 alone (0 unit control group, n=3 eyes). **B**: Confocal projection of optical z-sections demonstrating fluorescence after co-injection with chondroitin ABC lyase at the dose indicated. **C**: Confocal projection of optical z-sections demonstrating fluorescence after co-injection with heparinase III at the dose indicated.

Collagenase, hyaluronan lyase, chondroitin ABC lyase, and heparinase III were coinjected with AAV2.CBA.eGFP and retinal fluorescence was analyzed using confocal microscopy. As expected, the low dose of the AAV2 used (5×10^7^ viral particles) produced patchy, low intensity fluorescence in the retina, which is indicative of limited GFP expression (Figure 2). Collagenase treatment resulted in decreased GFP expression compared to AAV2 alone (Figure 2A). All of the other enzymes tested drove an increase in GFP expression (Figure 2A). The least effective was hyaluronan lyase, which nonetheless resulted in a 20-fold increase in retinal fluorescence at a dose of 100 units compared to the virus alone and extended the fluorescence signal to cells deeper in the retina and with more diverse morphology. The two other glycosidic enzymes, chondroitin ABC lyase and heparinase III, produced an even greater enhancement in retinal transduction. Chondroitin ABC lyase resulted in a 40 fold increase in fluorescence at 50 units, a 65 fold increase at 100 units, and a 150 fold increase at 200 units. Similarly, heparinase III treatment at 50 units resulted in approximately a 20 fold increase in AAV2 transduction, with a 50 fold increase at 100 units, and a 150 fold increase at 200 units. The chondroitin ABC lyase and heparinase III treatments (Figure 2B,C) resulted in a considerable increase in both the numbers and types of fluorescent cells compared to AAV2 alone (retinal images presented are collapsed over the z-axis).

### Enzyme dose–response curves

**Figure 3:**
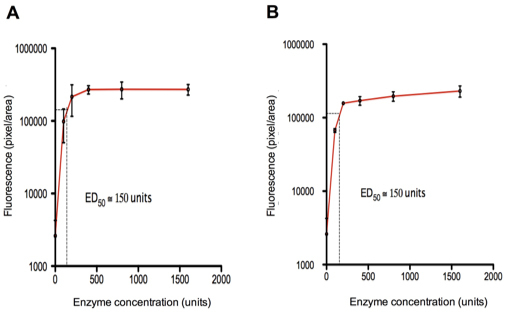
Dose–response curves showing treatment with **A**: Chondroitin ABC lyase, and **B**: Heparinase III. Values for fluorescence intensity represent the mean±SD (n=3 eyes). For both enzymes, the ED_50_ was between 100 and 200 units.

Next, a wider range of chondroitin ABC lyase and heparinase III concentrations was investigated to establish an optimal dose for each enzyme. Injections of six different concentrations were performed for each enzyme: 0 units (the PBS control), 100, 200, 400, 800, and 1,600 units. We quantified fluorescence intensity in ten samples per eye taken in a line across the width of the flatmounts to account for possible regional variations in cell transduction between central and peripheral retina (Figure 1B). Similar dose–response curves were observed for both enzymes that plateaued between 200 and 400 units and had an ED_50_ of between 100 and 200 units (Figure 3).

### Transduction is observed across the retinal depth

**Figure 4:**
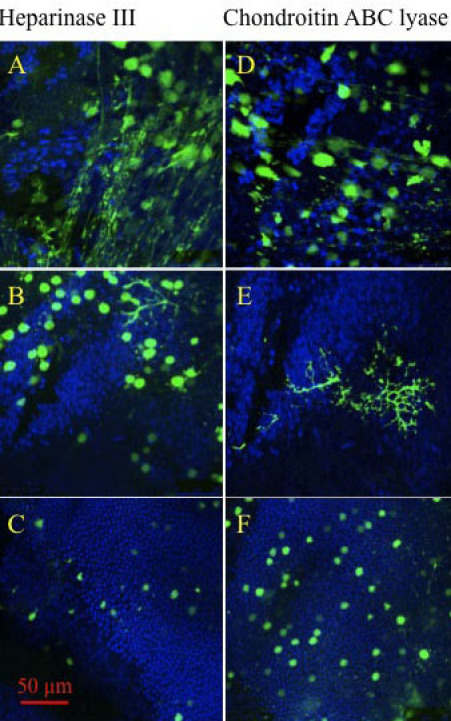
Sample confocal slices of retinas treated with AAV2.CBA.eGFP and heparinase III or chondroitin ABC lyase. GFP fluorescence and DAPI staining are merged and the images correspond to **A** and **D:** the inner retina, **B** and **E**: the mid retina, and **C** and **F**: the outer retina. Image sizes 238×238 µm.

The use of glycosidic enzymes also increased the depth of retinal transduction. Images of confocal slices from the inner, central, and outer retina following coinjection with heparinase III or chondroitin ABC lyase that demonstrate the different types of retinal cells transduced are shown in Figure 4. Marked fluorescence was seen in cell bodies and structures that resembled nerve fibers proximal to the vitreous. Deeper in the retina, fluorescence was observed in cells, including large arborizing neurons. Finally, patchy fluorescence was seen in the outer retina that co-localized with densely packed cell nuclei (observed by DAPI stain), suggesting that this fluorescence was within photoreceptor cell bodies.

**Figure 5:**
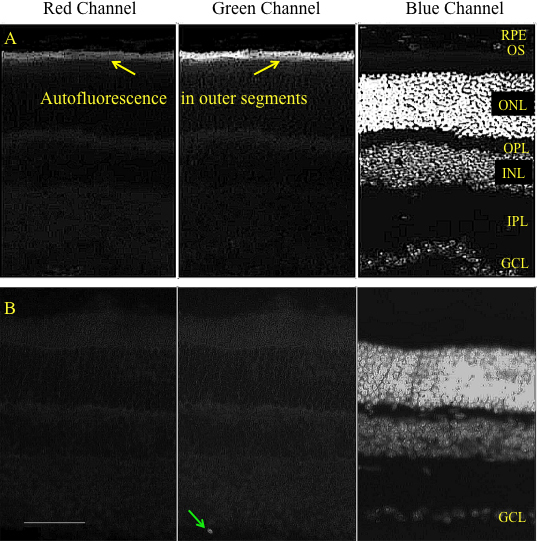
Representative cross-sectional micrographs show both untreated retinas and retinas two weeks after transduction with AAV2.CBA.eGFP, but with no enzyme treatment. **A**: Some sections of retina with no treatment (control) showed patchy autofluorescence in the photoreceptor outer segment layer. This could be distinguished from GFP fluorescence because it was observed in both red and green channels (see also Figure 6A, Figure 7D,E). **B**: Retina treated with AAV2.CBA.eGFP vector showed low transduction of the GCL. Texas Red filter is shown in the left panel; FITC filter is shown in the center panel; DAPI filter is shown in the right panel. Calibration bar 50 μm. Ganglion cell layer (GCL), inner plexiform layer (IPL), inner nuclear layer (INL), outer plexiform layer (OPL), Outer nuclear layer (ONL), Outer segments (OS) and Retinal pigment epithelium (RPE). Green arrow shows a retinal ganglion cell.

**Figure 6:**
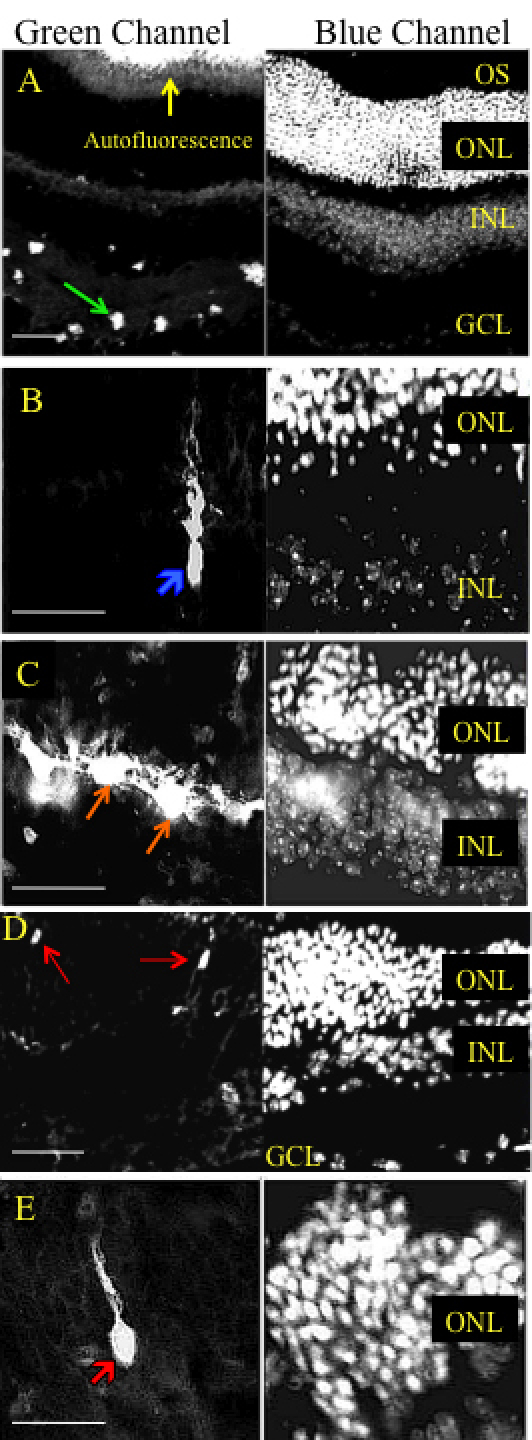
Representative cross sectional micrographs showing various retinal cell types transduced by AAV2.CBA.eGFP after treatment with chondroitin ABC lyase. **A**-**E**: A wide range of cell types were transduced in retinas treated with AAV2.CBA.eGFP vector and 200 units of chondroitin ABC lyase. GFP fluorescence was observed across the retinal layers, including cells with the anatomic location and morphology of; **A**: ganglion cells, **B**: Müller cells, **C**: INL cells, and **D**, **E**: photoreceptors. FITC filter is shown in the left panel; DAPI filter is shown in the right panel. Calibration bar 50 µm. Ganglion cell layer (GCL), inner nuclear layer (INL), outer nuclear layer (ONL), outer segments (OS). Green arrow shows a retinal ganglion cell. Blue arrow shows a Müller cell. Orange arrow shows cells in inner nuclear layer. Red arrow shows a photoreceptor cell.

**Figure 7:**
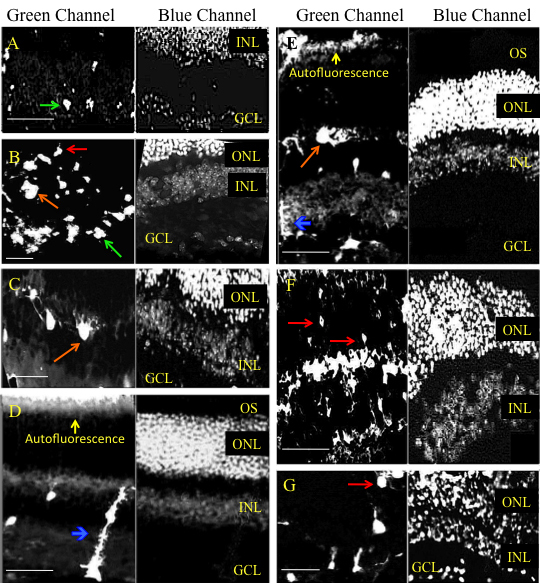
Representative cross sectional micrographs showing various retinal cell types transduced by AAV2.CBA.eGFP after treatment with heparinase III. **A**-**G**: After two weeks retinas treated with AAV2.CBA.eGFP vector and 200 units of heparinase III showed GFP expression across all retinal layers including cells with the anatomic location and morphology of (**A**-**E**, **G**) ganglion cells, (**A**-**G**) INL cells, (**D**, **E**) Müller cells, and (**F**, **G**) photoreceptor cells. FITC filter is shown in the left panel; DAPI filter is shown in the right panel. Calibration bar 50 µm. Ganglion cell layer (GCL), inner nuclear layer (INL), outer nuclear layer (ONL), outer segments (OS). Green arrow shows a retinal ganglion cell. Blue arrow shows a Müller cell. Orange arrow shows a cell in inner nuclear layer. Red arrow shows a photoreceptor cell.

To confirm the GFP expression in different retinal layers and to determine the retinal cell types transduced by the vector, we analyzed the fluorescence in histological cross sections of the retina. We co-injected mice with AA2-GFP and either 200 units of chondroitin ABC lyase (n=4 eyes) or heparinase III (n=4). The controls were injected with AAV2 and PBS (n=4). A double negative control with no injections (n=2) was included to show any endogenous retinal florescence. Our results confirmed that the use of glycosidic enzymes increased both the number and type of cells transduced across the retinal layers. Exemplar cross section images of the retina are shown in Figure 5, Figure 6, and Figure 7. Figure 5A shows the endogenous autofluorescence that was sometimes present in the layer containing the photoreceptor outer segments; this was observed in both the red and green channels, thereby distinguishing it from GFP fluorescence, which was observed in the green channel only. GFP expression was observed in occasional ganglion cells in eyes only treated with AAV2 without enzymes (Figure 5B).

Co-injection with chondroitin ABC lyase resulted in greatly enhanced GFP expression in the inner retina (Figure 6A) and there was some expression throughout the retinal layers, including Müller cells (Figure 6B), INL cells (Figure 6C), and ONL photoreceptors (Figure 6D,E). However, the pattern of transduction was patchy in most injected eyes. Similarly, co-injection of 200 units of heparinase III also produced a marked improvement in transduction across the retina (Figure 7A-G). GFP expression was most prominent in retinal ganglion cells (Figure 7A-E,G) and it was also detected in Müller cells (Figure 7D,E), in INL cells (Figure 7A-G), and in photoreceptors (Figure 7F,G). All histological sections appeared morphologically intact, so the enzymes did not produce an obvious disruption of retinal architecture.

### Analysis of retinal function in the presence of enzymes

**Figure 8:**
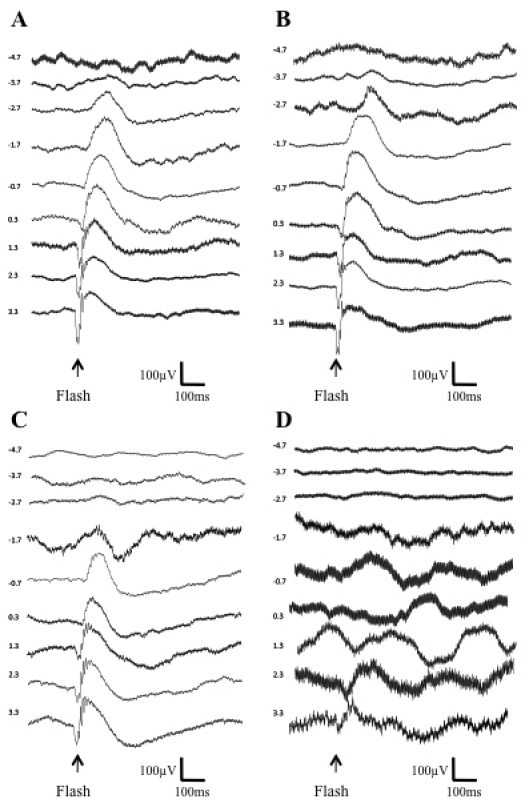
Electroretinograms (ERGs) following intravitreal delivery of AAV2.CBA.eGFP vector and heparinase III or chondroitin ABC lyase. **A**: Dark-adapted ERGs were intact following enzyme injection of heparinase III at 800 units. **B**: Dark-adapted ERGs were intact following enzyme injection of heparinase III at 1,600 units. **C**: Dark-adapted ERGs were intact following enzyme injection of chondroitin ABC lyase at 800 units. **D**: A higher dose of chondroitin ABC lyase (1,600 units) impaired retinal function. Records show average responses to repeated flash presentations in individual mice. Timing of each flash is indicated by the arrow, with flash intensity (in log10 μW.cm^−2^) shown to the left of each ERG trace.

To test the effect of glycosidic enzymes on retinal function, we recorded ERGs after digestion with 800 or 1,600 units of heparinase III and chondroitin ABC lyase (i.e., higher concentrations than required for maximal retinal transduction). ERGs were present and retained all major components (a-wave, b-wave, and oscillatory potentials) in mice treated with the AAV2.CBA.eGFP vector in conjunction with either 800 or 1,600 units of heparinase III or 800 units of chondroitin ABC lyase (Figure 8). However, a reduction in ERG amplitude was seen after intravitreal injections of 1,600 units of chondroitin ABC lyase.

## Discussion

AAV-mediated gene therapy holds great promise for treating or preventing visual loss in retinal dystrophies and other retinal conditions. Recently, remarkable progress has been made both in experimental approaches using animal models and in moving this technology into clinical trials. However, there is a need to improve transduction efficiency to broaden the applicability of AAV-mediated retinal gene therapy. There are several possible ways to improve AAV-mediated retinal transduction. The viral titer can be increased, but levels may be limited by the potential for immunogenicity [10]. Hybrid AAV can be constructed using the genome of one serotype, but using a packing capsid from another [1]. The AAV capsid can be mutated to enhance tropism and the efficiency of transgene expression; for example, recent experiments in which surface exposed tyrosine residues are mutated to decrease intracellular degradation show considerable promise [5]. A further approach is to tackle the physical barriers that prevent the AAV passing into and through the retina and this is the strategy explored in this study.

We hypothesized that after intravitreal injection, the viral particles become trapped in the extracellular matrices and by the cell surface proteoglycans found in the untreated vitreous, ILM, and neurosensory retina. These layers can form both physical and charge barriers that can limit the efficacy of trans-retinal penetration of macromolecules. Each barrier has a different GAG content and concentration and therefore leads to a variable level of viral entrapment [11-13]. HA and CSPGs in the vitreous; HA, CSPGs, and HSPGs in the ILM; and CSPGs and HSPGs in the retinal matrix could all inhibit viral transduction. However, electrostatic binding to the negatively charged GAGs is unlikely to impede vector penetration of the tissue because at a physiologic pH of 7.35, AAV with an isoelectric point ranging between 5 and 7 (depending on the serotype), would have an overall negative charge and would repel the anionic GAGs [14,15].

The major impedance is therefore likely to be due to the diffusion barrier caused by the vitreal, ILM, and retinal matrices (i.e., interphotoreceptor matrix), the external limiting membrane (ELM), and cell surface proteoglycans [16-18]. Possibly the most important barrier for these matrices is the inner limiting lamina (ILL), the basement membrane of Müller cells, which has pore channels varying in size from 10 to 25 nm (in rabbit retina) [17], whereas the AAV viral capsid has a total radius between 20 and 25 nm [19]. The intraocular movement of larger particles was investigated by Kamei et al. [18] who demonstrated that an intravitreally injected 70 kDa tissue plasminogen activator could not diffuse across the ILM in rabbits. Another study of antibodies delivered into the vitreous cavity of rhesus monkey found that Fab antibodies (48 kDa) diffused across the retina, but full-length antibodies (148 kDa) did not [20]. A further in vitro study by Jackson et al. [21] looked at the maximum size of the molecules capable of freely diffusing across human retina, or the retinal exclusion limit (REL), using FITC-dextrans of various molecular weights. They found the REL in human neuroretina to be 76.5±1.5 kDa (6.11±0.04 nm). Furthermore, they observed only moderate interspecies variations in animal studies including those that for pigs, cattle, and rabbits (60±11.5, 78.5±20.5, and 86±30 kDa, respectively), which are commonly used to model human disease. Interestingly, they found that in human retina, the inner and outer plexiform layers formed the sites of highest resistance to diffusion, which were even greater than the ILM. Larger molecules were capable of crossing the retina, although the rate of diffusion was much reduced.

The wild-type murine retinas show very limited inner retinal penetration by intravitreally delivered AAV vectors. This lack of permeability into the outer retina is not dependent on AAV serotype including types 2, 5, 7, 8, and 9 as shown in a study by Lebherz et al. [22]. The researchers showed that after intravitreal injection into mouse eyes, only AAV2 and AAV8 were able to transduce retinal ganglion cells and AAV2, 8, and 9 transduced occasional Müller cells. None of the other vectors tested demonstrated significant retinal expression after intravitreal injection. Interestingly, viral particles spread into the outer retina and RPE after intravitreal delivery in degenerate retinas compared to their accumulation at the ILM in normal rat retina [23]. Immunohistochemistry showed changes in the architecture of the ILM, which are likely to underlie the increased viral transduction in diseased tissue. Similarly, the lack of retinoschisin (Rs1) causes a marked change in the permeability of the retina to AAV vectors, as serotypes including 2, 5, and 8 were able to penetrate all retinal layers of Rs1-KO mice and even to transduce the retinal pigment epithelium from the vitreous [24].

Previous studies have investigated the disruption of extracellular barriers in ocular gene therapy. Gruter et al. [25] investigated the effects of enzymatic digestion on the barrier properties of the interphotoreceptor matrix (IPM) following subretinal injections of lentiviral vector aimed at transfecting photoreceptors. Digestion of the IPM with neuraminidase X, and to a lesser extent with chondroitinase ABC (digesting mainly cone matrix), resulted in some improvement in photoreceptor transduction. A recent publication by Dalkara et al. [26] demonstrated that AAV2 localized at the ILM after intravitreal injection and that digestion of the ILM with pronase E, a nonspecific protease, qualitatively improved retinal transduction. It has also been shown that degradation of intra-retinal GAGs with chondroitin ABC lyase promotes the migration and integration of stem cells into degenerating retina [27]. Consistent with other publications [1,26] we observed that intravitreally delivered AAV2 alone leads to a low retinal transduction rate. Collagenase digestion decreased retinal transduction efficiency, but chondroitin ABC lyase, heparinase III and, to a lesser extent, hyaluronan lyase increased expression of the viral reporter gene. It is likely that the glycosidic enzymes promote viral transduction by breaking down vitreal and retinal extracellular matrices, thus increasing the size of the matrix pores and allowing AAV to move across these barriers with more ease.

The differences observed in the effects of these glycosidic enzymes might be explained by regional variations in GAG content and concentration. The highest concentration of HA is in the vitreous. However, as the vitreous contains such a dilute extracellular matrix, it may not greatly impede the movement of AAV particles, so digestion of the vitreous HA network with hyaluronan lyase may have a modest effect, as observed in our study. The ILM has been shown to be a barrier to AAV entering the retina [26] and a major barrier within this structure is likely to be the ILL, where the predominant proteoglycans are thought to be heparan sulfate proteoglycans.

Therefore, the major effect of heparinase III digestion is likely to be an increase in permeability of the ILL. However, a recent study by other researchers in our laboratory has demonstrated that ILM also contains chondroitin sulfate (CS) and dermatan sulfate (DS) GAGs (unpublished observation). This could explain the equally robust effect of chondroitin ABC lyase in increasing retinal transduction by its action on the ILL. Furthermore, both CSPGs and HSPGs are abundant in the other retinal layers, including the nerve fiber layer, the inner and outer plexiform layers, and in the interphotoreceptor matrix. Hence, digestion with chondroitin ABC lyase or heparinase III could make any of these retinal layers more porous and thus improve the trans-retinal penetration of the viral vector.

It is of interest to note that heparinase III was effective at increasing retinal transduction, as infection of cells by AAV2 has been shown to be heparan sulfate dependent [28]. However, cell surface heparan sulfate proteoglycans are not always required for internalization of AAV2 [28,29], so their removal by heparinase III digestion may not have compromised the ability of retinal cells to take up the AAV2. Alternative explanations are that the digestion of retinal heparan sulfates was incomplete, or that new cell surface heparan sulfate proteoglycans were synthesized, permitting AAV2 entry into the retinal cells after the heparinase III digestion (which is likely to have a short duration of action) had disrupted extracellular barriers, facilitating movement of the virus particles into and within the retina.

The use of intravitreally-delivered glycosidic enzymes for other purposes has been investigated. Hyaluronidases and chondroitinase have been tested in animal models for pharmacological vitreolysis without any reported adverse effects [30-32]. Furthermore, highly purified ovine hyaluronidase VitraseTM (ISTA Pharmaceuticals, Irvine, CA) has been used in clinical trials to aid the dispersion of vitreous hemorrhage [33]. We investigated whether the enzymes had functional effects on the retina, by measuring ERGs, and found that light responses appeared intact after digestion with 800 units of heparinase III or chondroitin ABC lyase i.e., at a dose that was considerably higher than the 200 units required for maximal retinal transduction. It is still possible that these enzymes damage inner retinal function at doses of 800 units or less, but it is of note that in experiments with pronase E, measuring ERGs was a more sensitive indicator of retinal damage than measuring VEPs [26]. Nevertheless, an important element of future work will be to focus on the more extensive assessment of any acute and/or chronic effects these glycosidic enzymes may have on retinal function.

Taken together, our data suggest that heparinase III or chondroitin ABC lyase greatly increased retinal transduction by intravitreal AAV2. This approach will be useful for experimental gene transfer into the retina using rodent models and may broaden the applicability of AAV-mediated gene therapy for treating human disease.
